# Tricky with Heat and Salt: Soil Factors, Thermotaxis, and Potential for Heat–Saline Agar Trapping of *Strongyloides* Larvae

**DOI:** 10.3390/biology14050559

**Published:** 2025-05-16

**Authors:** Nuttapon Ekobol, Sirintip Boonjaraspinyo, Chatanun Eamudomkarn, Thidarut Boonmars

**Affiliations:** 1 Department of Parasitology, Faculty of Medicine, Khon Kaen University, Khon Kaen 40002, Thailand; nattek@kku.ac.th (N.E.); chatea@kku.ac.th (C.E.); 2 Department of Community, Family and Occupational Medicine, Faculty of Medicine, Khon Kaen University, Khon Kaen 40002, Thailand; sboon@kku.ac.th

**Keywords:** *Strongyloides*, infective larvae, inactivation, soil types, noxious heat, thermotaxis, horizontal migration, vertical migration, larval trapping

## Abstract

The viability and host-seeking behavior of *Strongyloides* larvae are significantly influenced by soil conditions, emphasizing the critical role of environmental control in disease management. This is particularly relevant given the growing concerns about drug resistance resulting from mass chemotherapy or the use of chemical nematicides. *Strongyloides stercoralis* was effectively inactivated by exposure to 50 °C for both 12 and 24 h (long-term exposure). *Strongyloides ratti* was inactivated by 50 °C for 20 min (short-term exposure), 9% saline for 50 min, and a combination of 4% saline and 40 °C for 50 min. The combined treatment successfully inactivated *S. ratti* in four soil mediums using 5% saline at a central temperature of 40 °C. Thermotaxis responses to noxious heat revealed attraction at 40 °C, increased localized searching at 45 °C, and complete inactivation at 50 °C. Larvae migrating within agar at 45 °C were more readily inactivated. Long-range heat attraction at 5 cm resulted in the inactivation of up to 50% of incoming larvae; however, heat-high concentration saline traps at 3 cm distance proved ineffective. Thermal–saline agar trapping demonstrated promise for larval removal in sand, loam, and laterite soils. This method offers a promising approach to larval removal while minimizing hazards to non-target organisms.

## Simple Summary

Soil factors significantly influence *Strongyloides* infective larvae activity and viability. While heat, saline, and pH are commonly manipulated for soil environmental control, their effectiveness in achieving larval inactivation is inconsistent. This study used *S. stercoralis* and *S. ratti* to assess mortality and inactivation under heat, saline, and pH exposure, both directly and through soil media. Larval response to noxious heat and high-concentration saline was examined, and the effectiveness of heat–saline larval trapping was determined. This research provides insights into combined stressor inactivation, larval behavior under noxious heat, and the potential of heat–saline agar trapping.

## 1. Introduction

Soil is a critical component of the human environment, providing essential services such as nutrient cycling, moisture retention, and biodiversity support, all of which contribute to human health [1,2,3]. However, soil can also harbor pollutants and infectious agents, poising a significant risk of disease transmission [4,5]. Among soil-transmitted helminth infections, strongyloidiasis, caused by *Strongyloides stercoralis*, is a major public health concern, estimated to infect approximately 614 million people worldwide [6]. This often asymptomatic and neglected infection exhibits a wide range of clinical presentations, from acute infection to mild or subclinical chronic infection, and severe disease (hyper infection syndrome and disseminated disease) [7,8,9]. While severe strongyloidiasis, characterized by high mortality rates, is relatively rare and primarily observed in immunocompromised individuals (e.g., those with human T-lymphotropic virus type 1 infection, malignancy, or alcoholism, or those undergoing immunosuppressive therapy), chronic mild or asymptomatic infections are far more common [10,11,12,13,14,15]. Chronic strongyloidiasis, though often overlooked, can have significant health implications, particularly for vulnerable populations. Pregnant women and young children may experience nutritional deficiencies and developmental delays due to infection [16,17]. Agricultural workers and migrant populations, who have frequent contact with soil, are also at increased risk, with prevalence rates reaching up to 40% [18]. Current strategies for the prevention and control of strongyloidiasis primarily focus on promoting personal protective equipment (e.g., shoes and gloves) and implementing preventive chemotherapy [6,19,20]. Regular deworming programs are also effective, but the widespread use of anthelmintic drugs raises concerns about the development of drug resistance [21,22]. Integrating environmental control measures is crucial for interrupting transmission. Improving sanitation through safe waste disposal systems can also limit the spread of larvae [23,24]. Soil treatment to eradicate parasitic nematodes has been investigated, and nematicides are widely used in agriculture for controlling plant parasites. However, there is potential toxicity and development of resistance, particularly regarding their effects on animal parasitic nematodes [25,26]. Biocontrol using nematophagous fungi has shown promise in reducing plant and animal parasitic nematode populations, but large-scale field studies of strongyloidiasis are needed to validate its efficacy [27,28]. The geographic distribution of strongyloidiasis, as well as the occurrence of helminths in soil and prevalence of infection in humans and animals, is influenced by various soil factors, including soil type, temperature, moisture, organic matter content, and pH [29,30,31,32]. Heat treatment for human fecal pit soil required 60 °C and 60 min of exposure to inactivate *Strongyloides* larvae [32]. Only strong acid (pH 1.2) and strong base (pH 10.2) showed effective inactivation of the larvae from wastewater [33]. Saline was not recognized as an effective inactivation factor and required saturate solution with 30 min contact time to inactivate filariform larvae [34]. Temperature is a critical factor for both developmental choices and thermotaxis. Ambient temperatures below 34 °C induce the post-parasitic L1 stage to develop into indirect life cycle (free-living adult), while temperatures above 34 °C induce the direct life cycle (infective L3 larvae) [35]. The starting temperature of 23 °C is the switching point for *S. stercoralis* to select positive or negative thermotaxis [36]. Both types of migration increase the chance of finding a worm-blood host. Most *Strongyloides* thermotaxis studies have been conducted on a horizontal plane, lacking information on vertical migration, which is influenced by gravity and often performed on hard thermotaxis agar (surface migration), unlike the soil environment that offers surface, within-soil body, and mixed types of migration. Moreover, data about the thermotaxis of *Strongyloides* in response to noxious heat are still undiscovered. The available methods to eliminate parasites in soil with heat include steaming treatment and soil solarization [37]. Inactivation with 60 °C steaming throughout 10 cm soil depth takes place, maintaining this temperature for at least 30 min. Covering the soil surface with a plastic film to trap solar radiation can maintain soil temperature around 50 °C (inconsistent heat), which can show effective inactivation for a 5 cm soil depth but requires 5 days of solarization. However, both methods apply high temperatures that may be harmful to useful organisms and are less effective at inactivating parasites in deep soil. Therefore, this study aimed to conduct laboratory experiments to evaluate the nematocidal effects of specific soil factors (noxious heat, saline, and soil texture) on *Strongyloides* infective larvae.

## 2. Materials and Methods

### 2.1. Inactivation of Strongyloides stercoralis Infective Larvae by Temperature and pH

Residual fecal specimens that tested positive for *Strongyloides* by the formalin-ethyl acetate concentration technique (FECT) in a primary study involving monk participants [38] were utilized for the Harada–Mori filter paper strip culture technique. The primary study, which investigated parasitic infections and associated factors in monks, was conducted from July 2021 to December 2021. In that study, approximately 10 g of fecal specimen were collected from each participant. A total of 3 g of each specimen was processed using FECT, and 2 g was used for the agar plate culture technique. All remaining specimens were stored in an ice box. On the day of processing, specimens testing positive for *Strongyloides* were pooled, diluted with normal saline, and thoroughly mixed before the Harada–Mori filter paper strip culture techniques were performed. On day 4, the culture water containing mixed-stage larvae was discarded, and infective larvae were collected the following day. The washing steps were performed using refrigerated centrifuge at 3000 rpm and 4 °C for 10 min with maximum acceleration and no end brake. The supernatant was discarded, and the samples were washed twice with distilled water. The washed larvae were examined under a compound microscope to confirm their activity, and serial dilutions were performed to achieve a concentration of approximately 50 larvae/10 μL.

The pH buffers were prepared using distilled water, hydrochloric acid, and sodium hydroxide to achieve pH levels of 3, 5, 7, 9, and 11. The incubation temperatures were set at 15 °C and 25 °C using an icebox, and at 35 °C, 40 °C, and 50 °C using a water bath. A 10 μL volume of larvae was mixed with 40 μL of pH buffer for pH incubation, and with 40 μL of distilled water for temperature incubation in 96-well plates. The incubation temperature was monitored and recorded every hour. Larval movement and mortality were assessed after 12 and 24 h of incubation using propidium iodide staining and observation with a confocal stereomicroscope (Appendix A.1).

### 2.2. Inactivation of Strongyloides ratti Infective Larvae by pH, Temperature, and Saline Solution

The culture water was collected on day 5 (Appendix A.2), washed twice with distilled water using refrigerated centrifugation, and then diluted to achieve a final concentration of 50 larvae/μL. Buffers were prepared at pH values of 3, 5, 7, 9, and 11. Saline solutions were prepared ranging from 1% to 10% (increasing by 1% for each setup), as well as at 20% and saturated saline solution (approximately 35 g of sodium chloride per 100 mL), all at room temperature. Two microliters of infective larvae suspension were added to a 200 μL tube. Then, 8 μL of pH or saline buffer was added along the inner wall of the tube, while 8 μL of distilled water was used for the temperature test. The tube racks were gently tapped to facilitate mixing of the solution. Test tubes were placed in EVA foam to enable floatation in a water bath, which was maintained at temperatures of 40, 45, 50, 55, 60, 65, and 70 °C for the temperature inactivation test. Inactivation of infective larvae was measured after exposure times of 10, 20, 30, 40, 50, and 60 min. The optimal pH, temperature, and salinity conditions were used for the further combined infective larvae inactivation test.

### 2.3. Measurement of the Inactivation of S. ratti Infective Larvae

After reaching the exposure time, the entire volume of the larval solution was aspirated and dropped onto a glass slide. The larvae were then stimulated using a 25G needle. Video footage was recorded for 30 s using a stereomicroscope (Olympus SZ51). The videos were processed using iMovies to crop and invert the video colors, creating a white background. They were then labeled for counting with Keynotes as active larvae (green dots), inactive larvae (red dots), or artifact (purple dots).

### 2.4. Soil Heat Conduction and the Inactivation of S. ratti Infective Larvae

Soil samples, including sand, loam, laterite, and clay, were collected from different areas of Khon Kaen University. The soil texture was confirmed using the ribbon method and the shaking test [39,40]. For the ribbon method, approximately 25 g of soil was wetted in the palm until moldable, and the ability to form a stable ball was assessed; failure to form a ball indicated sand. If a ball formed, it was pressed between the thumb and forefinger to create a ribbon, the length of which determined the classification: less than 2.5 cm was loam, between 2.5 cm and 5 cm was clay loam, and more than 5 cm was clay; the feel of the wetted soil (gritty or smooth) was further used to classify these categories with sandy or silty components. For the shanking test, a wetted soil sample was formed into a patty (8 cm diameter, 1.5 cm thick), placed on the palm, and shaken horizontally; a rapidly developing shiny surface indicated silt, while a dull surface indicated clay, which was confirmed by blending the patty and observing if the surface remained dull; finally, the dried patty’s structural integrity was assessed, with blistering and dust upon rubbing suggesting silt and firm, dust-free consistency suggesting clay. The samples were then sun-dried for 8 h, crushed into small pieces, and sieved through a 1 mm mesh to obtain fine soil particles. The filtered soil was then oven-dried at 105 °C for 24 h [41]. Dried soil samples were placed into No. 2 plastic cups, with each cup containing 50 g of soil. A NTC (negative temperature coefficient) temperature sensor was positioned at the center of each cup. The soil containers were placed in a water bath set to 45 °C, 50 °C, 55 °C, and 60 °C. The central temperature of each sample was recorded at 10, 20, 30, 40, 50, and 60 min. A 2 μL larval solution was mixed with 8 μL of distilled water or an optimized saline concentration in a 200 μL tube, which was then immersed in soil and incubated in water bath set to achieve a central temperature of 50 °C. The inactivated larvae were assessed at time points of 10, 20, 30, 40, 50, and 60 min.

### 2.5. Response of S. ratti Infective Larvae to Hazardous Heat Exposure

The heating system consisted of a digital PID (proportional integral derivative) controller, a solid-state relay with a heat sink, an infrared radiation heater, and a thermocouple heat sensor. The thermotaxis chamber was constructed from a clear acrylic sheet with a thickness of 3 mm. Two types of chambers were used: an open chamber measuring 1 × 20 × 1.7 cm and a closed chamber measuring 1 × 20 × 1 cm. The sliding-stage thermotaxis assay was assembled using a clear acrylic sheet (5 mm thick) with dimensions of 45 × 20 cm. A socket for the thermotaxis chamber was created from EVA foam, forming a rectangular area measuring 2.5 × 20 cm, and was fitted with a transparent scale bar. Steam indicator tape was applied to mark the heater positioning area. Two metallic arms were secured with strong double-sided adhesive tape to hold the heater in place for the vertical assay. Three concentrations of agar were prepared for the thermotaxis experiment (Appendix A.3).

The horizontal and vertical migration of infective larvae in response to hazardous heat was examined using different chamber setups: an open chamber with hard agar to assess surface migration, a closed chamber with semi-solid agar to evaluate migration within the medium, and an open chamber with semi-solid agar to observe mixed migration patterns. An NTC temperature sensor was installed in the thermotaxis chamber. Thermotaxis agar was poured and allowed to set and dry for 20 min for hard agar and 30 min for semi-solid agar. In the closed chamber, sealant agar was applied to seal the base before pouring the semi-solid agar, which was allowed to set for 1 h. A washed larvae solution was prepared at a concentration of approximately 30 larvae/μL for group migration tests and 1 larvae/μL for single-worm migration assays. A radiant infrared heater, thermocouple sensor, NTC temperature sensor, and thermotaxis agar chamber were mounted onto the sliding stage.

The radiant heater was switched on to maintain the temperature at the starting area at 40 °C, 45 °C, and 50 °C for at least 5 min before the observation began. The larvae solution was dropped at the starting point (0 cm area), where it was placed on the agar surface; tilted at 45° and injected into the agar media; and inserted parallelly for surface, mixed, and inside media migration assays. The video was recorded for 10 min (starting from when the water evaporated) using a stereomicroscope with a 0.8× objective magnification. This method was repeated 10 times for the single-worm assay and 3 times for group migration assay. The stereomicroscope was flipped 90° to record videos for the vertical plane migration assay. The video was processed using iMovies to crop it and convert it to black and white color. For single worm migration, the worm’s position was marked and drawn every 5 s. For group migration, the starting and ending positions were marked.

### 2.6. Long-Range Heat Attraction of S. ratti Infective Larvae and Heat-High Concentration Saline Trapping

Infective larvae were placed 5 cm from the heater on hard thermotaxis agar (both horizontal and vertical orientation), with the heater set to 40 °C, 45 °C, or 50 °C. Inactivation percentages at 50 °C were then determined using groups of approximately 30 larvae.

The attraction of larvae to saline solutions (5%, 10%, 20%, and saturated) under thermal stimulation (40 °C) was investigated on the horizontal plane. Infective larvae were placed 3 cm from the heater, and saline solutions were dispensed to create a 3 mm-wide area adjacent to the heater. The percentage of larvae trapped in the saline was determined by observing group migration towards this 3 mm-wide saline “well” on hard agar. To create the well, the agar surface was cut with a stainless-steel spatula, the agar base was sealed with 2 drops of warm agar, and the well was then filled with the warm saline solution. Video recordings (15 min) were used to track the migration of individual worms and to quantify the number of worms trapped within the saline well (5%, 10%, 20%, and saturated saline), thus determining the trapping percentage. The temperature at the starting points (5 cm and 3 cm from the heater) was measured using an infrared thermometer.

### 2.7. Thermal Trapping and Removal of S. ratti Infective Larvae from Soil with Saline Agar Cuboid

Dried soil samples (50 g dry weight) were rehydrated. Distilled water was added: 5 mL for sand, loam, and laterite soils (equivalent to 10% *w*/*w* moisture content) [42], and 20 mL for clay soil (equivalent to 40% *w*/*w* moisture content). Semi-solid agar cubes were prepared using acrylic molds (1 × 2 × 1 cm). The lower portion of acrylic box was immersed in sealant agar and then placed on glass slide to create leak-proof base. A 5% (*w*/*v*) salt semi-solid agar solution was poured into each mold and allowed to solidify for 40 min. To prepare soil migration chambers, the base acrylic box was placed on a 5 cm diameter glass Petri dish and the sealant agar was poured to secure it. Each chamber was filled with appropriate moist soil, ensuring the soil was neither compacted nor contained air pockets. Four soil-filled chambers were stacked vertically. The topsoil surface of the uppermost chamber was covered with two layers of gauze (2.5 × 1.5 cm). Warm distilled water (40 °C) was then added to the top of the gauze: 500 μL for sand, 300 μL for loam and laterite, and 100 μL for clay. The stacked soil chambers were warmed from above using an infrared heater with 3 cm gap from the uppermost soil box, maintaining the topsoil surface temperature at 40 °C, monitored with an infrared thermometer. Infective larvae (100 μL suspension containing 30 larvae/μL, for a total of 3000 larvae) were placed on the soil surface of the bottommost chamber. All junctions between the stacked chambers were sealed with Parafilm^®^ to prevent larval escape [43]. Agar cuboid covering the gauze was collected along a 1 h heat attraction period; the first agar cuboid was removed after 20 min, and subsequent cuboids were removed every 15 min for a total of four collections. To extract larvae from the topsoil, a Baermann technique was employed. The soil in the top chamber was pulled out, wrapped in a 3 mm-thick cotton sheet, and incubated in warm distilled water (40 °C) for 30 min. The collected agar cuboids were melted on a hot plate (150 °C for 2 min or until homogenous) and poured onto two glass slides. The Baermann extract was centrifuged (3000 rpm, 10 min, 4 °C, maximum acceleration, and no end brake). The supernatant was discarded, the resulting 5 mL sediment was retained, and 1 drop of 1% iodine solution was added. The number of infective larvae in both the Baermann extract and melted agar samples was determined using a compound microscope at 40× magnification.

### 2.8. Statistical Analysis

Data on *Strongyloides* infective larvae mortality, inactivation, and larval trapping were analyzed and are presented as mean ± standard error of the mean (SEM). To determine the effects of temperature, pH, saline concentration, and soil texture on larval mortality, inactivation, and trapping, statistical analysis was performed on data from three independent replicates using one-way analysis of variance (ANOVA). These analyses were conducted using SPSS version 29.0.1.0 (IBM Corp., Armonk, NY, USA) under a KKU network license. Statistical significance was defined as a *p*-value of less than 0.05. Where ANOVA revealed significant differences among groups, post hoc comparisons were conducted using Tukey’s honestly significant difference (HSD) test to identify specific pairwise differences.

## 3. Results

### 3.1. Strongyloides stercoralis Infective Larvae Exhibited Susceptibility to 50 °C but Resistance to Varying pH

**Figure 1:**
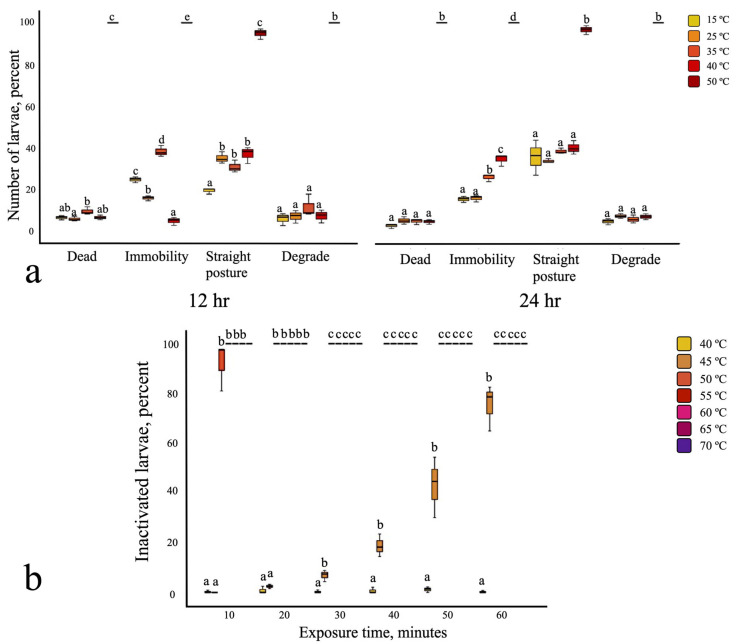
Effect of heat exposure on *S. stercoralis* and *S. ratti*. (**a**) Long-term exposure of *S. stercoralis* iL3 at 12 and 24 h at 15 °C to 50 °C. (**b**) Short-term exposure of *S. ratti* iL3 at 40 °C to 70 °C. All experiments were conducted in three replicates. Different letters (a, b, c, d, e) indicate statistically significant differences between groups based on the Tukey HSD post hoc test.

The effects of prolonged incubation at various temperature on the mortality of infective larvae were assessed by evaluating four parameters: immobility, straight posture, degradation of esophageal–intestinal structure, and propidium iodide (PI) fluorescence. Incubation occurred at 50 °C for both 12 and 24 h, resulting in 100% mortality using PI fluorescence analysis, immobility, and degrade esophageal–intestinal structure, while we observed only 95% in straight posture (Figure 1a). Incubation at temperatures of 15 °C, 25 °C, 35 °C, and 40 °C had a minimal effect on larval mortality. Infective larvae exhibited high resistance to varying pH levels (pH 3, pH 5, pH 7, pH 9, and pH 11), with mortality remaining below 5% at both time points (Figure S1a).

### 3.2. Strongyloides ratti Infective Larvae: Susceptibility to Heat, Salinity, and Combined Stress

**Figure 2:**
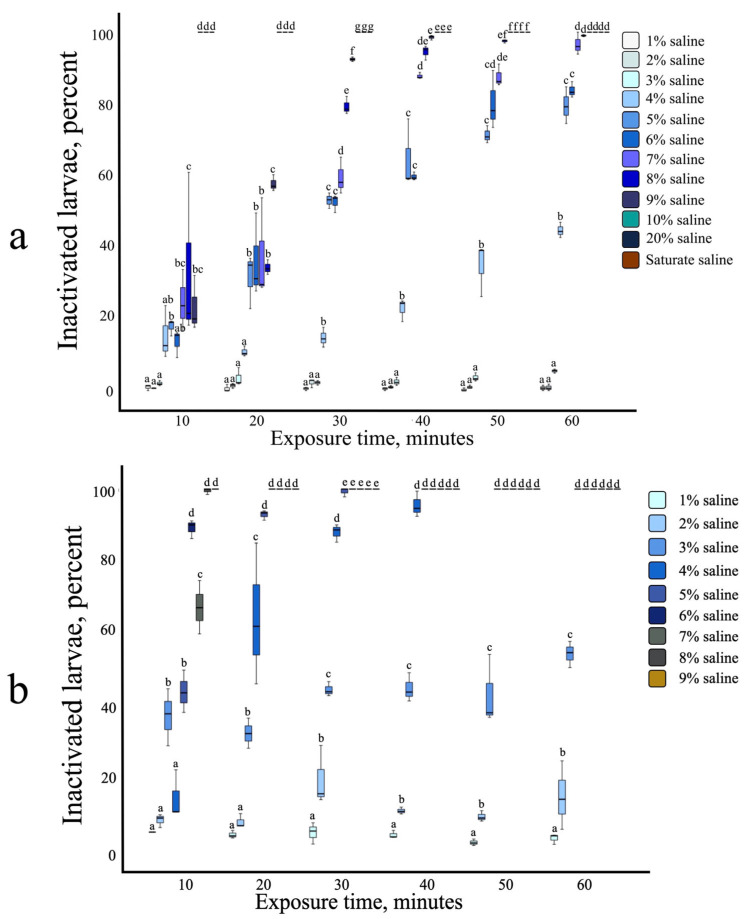
Effect of saline and saline–heat combination on *S. ratti* infective larvae: (**a**) Various saline concentration from 1% saline to saturated saline. (**b**) Saline concentration from 1% to 9% at 40 °C. All experiments were conducted in three replicates. Different letters (a, b, c, d, e, f, g) indicate statistically significant differences between groups based on the Tukey HSD post hoc test.

Incubation at 50 °C for 20 min resulted in 100% inactivation of infective larvae (Figure 1b). Complete inactivation with saline alone required exposure to 9% salinity for 50 min (Figure 2a). The combination of 4% salinity and 40 °C achieved 100% larval inactivation within 50 min (Figure 2b). *S. ratti* infective larvae exhibited low susceptibility to pH levels ranging from 3 to 11 after 60 min of incubation (Figure S1b).

### 3.3. The Effect of Soil–Heat Conduction and Combined Heat–Salinity Treatment on S. ratti Larval Inactivation

**Figure 3:**
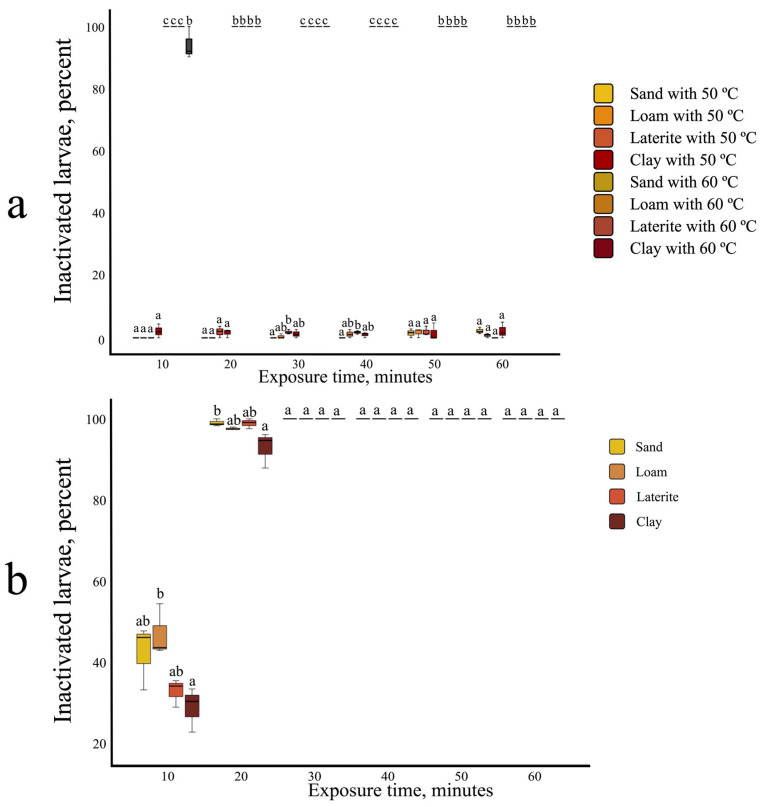
Effect for heat inactivation through soil medium. (**a**) Water bath set at 50 °C and 60 °C (central temperature 45 °C compare with central temperature 50 °C). (**b**) Combined effect of 5% saline at water bath temperature 45 °C (central temperature 40 °C). All experiments were conducted in three replicates. Different letters (a, b, c) indicate statistically significant differences between groups based on the Tukey HSD post hoc test.

For all four soil types, water bath temperatures of 45 °C and 60 °C were required to reach a central temperature of 40 °C and 50 °C within 20 min. The effect of soil–heat conduction on larval inactivation was assessed using water baths set to 50 °C (resulting in an approximate central temperature of 45 °C) and 60 °C. After a 10 min exposure, sand, loam, and laterite soils showed 100% larval inactivation (Figure 3a). Clay soil, however, reached only 94.13% inactivation at 10 min and required a 20 min incubation for complete larval inactivation. For the combined stress inactivation experiment, a water bath set to 45 °C (resulting in a central temperature of 40 °C) was used in conjunction with 5% salinity. This combined treatment achieved 100% larval inactivation after 30 min for all four soil types (Figure 3b).

### 3.4. Thermal Response in the Horizontal Plane

**Figure 4:**
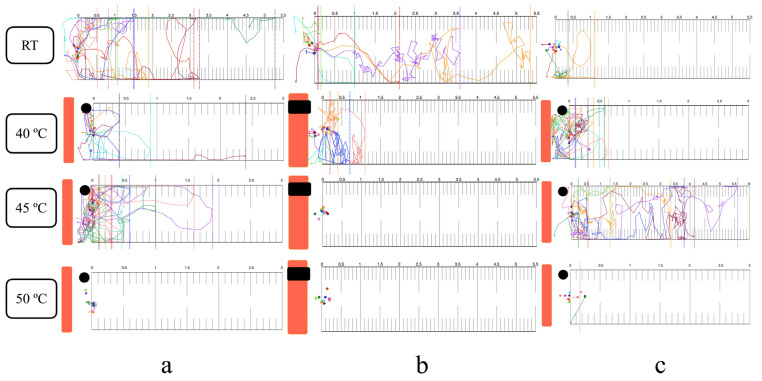
Single-worm horizontal migration response to noxious heat: (**a**) Surface route with hard thermotaxis agar and open chamber. (**b**) Within-agar route with semi-solid thermotaxis agar and closed chamber. (**c**) Mixed migration route with semi-solid thermotaxis agar and open chamber. Orange bars: radiant heater. Black circles or rectangles: NTC temperature sensors. Colored dots and arrows: larval starting positions and migratory tracks. Dashed lines: maximum migration distance from heater. All experiments were conducted in ten replicates.

**Figure 5:**
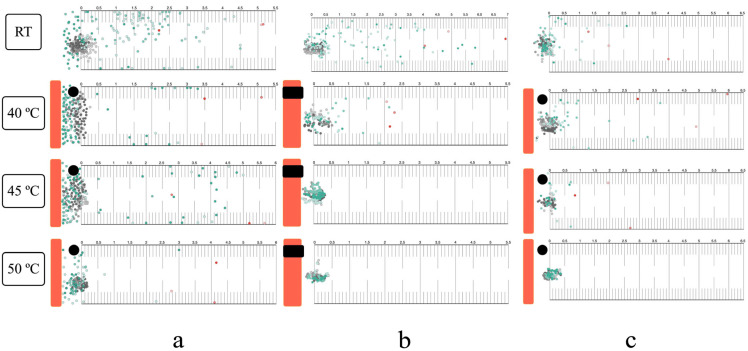
Group horizontal migration response to noxious heat: (**a**) Surface route with hard thermotaxis agar and open chamber. (**b**) Within-agar route with semi-solid thermotaxis agar and closed chamber. (**c**) Mixed migration route with semi-solid thermotaxis agar and open chamber. Orange bars: the radiant heater. Black circles or rectangles: NTC temperature sensors. Gray dots: starting position. Green dots: position at the end of observation. Red dots: the longest migratory distance of each replicate. All experiments were conducted in three replicates.

Infective larvae exhibited localized searching at room temperature across all migration assays (surface, within-agar, and mixed, both single and group) (Figure 4). Positive thermotaxis was observed in single larvae at 40 °C, regardless of migration route. At 45 °C, surface and mixed migration showed increased localized searching and positive thermotaxis, while within-agar migration resulted in immediate inactivation (Figure 4b). Complete inactivation occurred at 50 °C in all assays. Group migration responses mirrored those of single larvae (Figure 5 and Figure S2).

### 3.5. Thermal Response in the Vertical Plane

**Figure 6:**
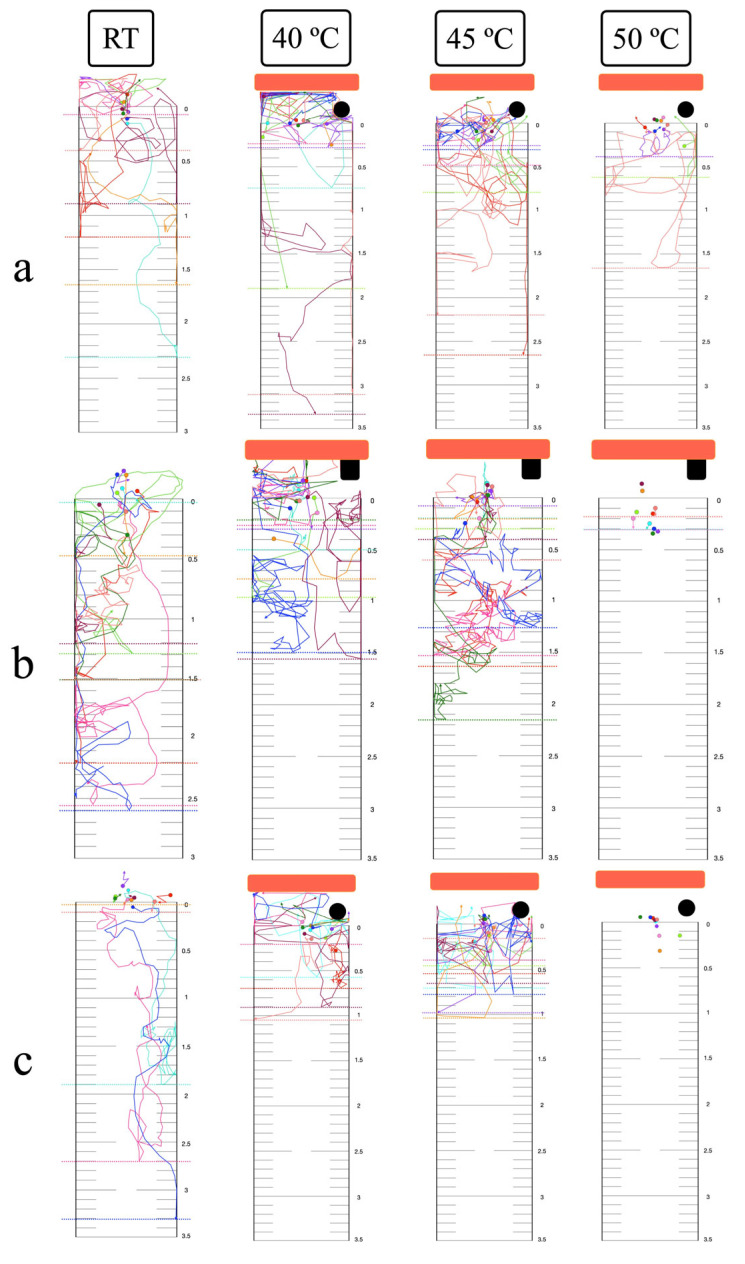
Single-worm vertical migration response to noxious heat: (**a**) Surface route with hard thermotaxis agar and open chamber. (**b**) Within-agar route with semi-solid thermotaxis agar and closed chamber. (**c**) Mixed migration route with semi-solid thermotaxis agar and open chamber. Orange bars: the radiant heater. Black circles or rectangles: NTC temperature sensors. Colored dots and arrows: larval starting positions and migratory tracks. Dashed line represents the longest downward migratory distance of a single-worm assay. All experiments were conducted in ten replicates.

**Figure 7:**
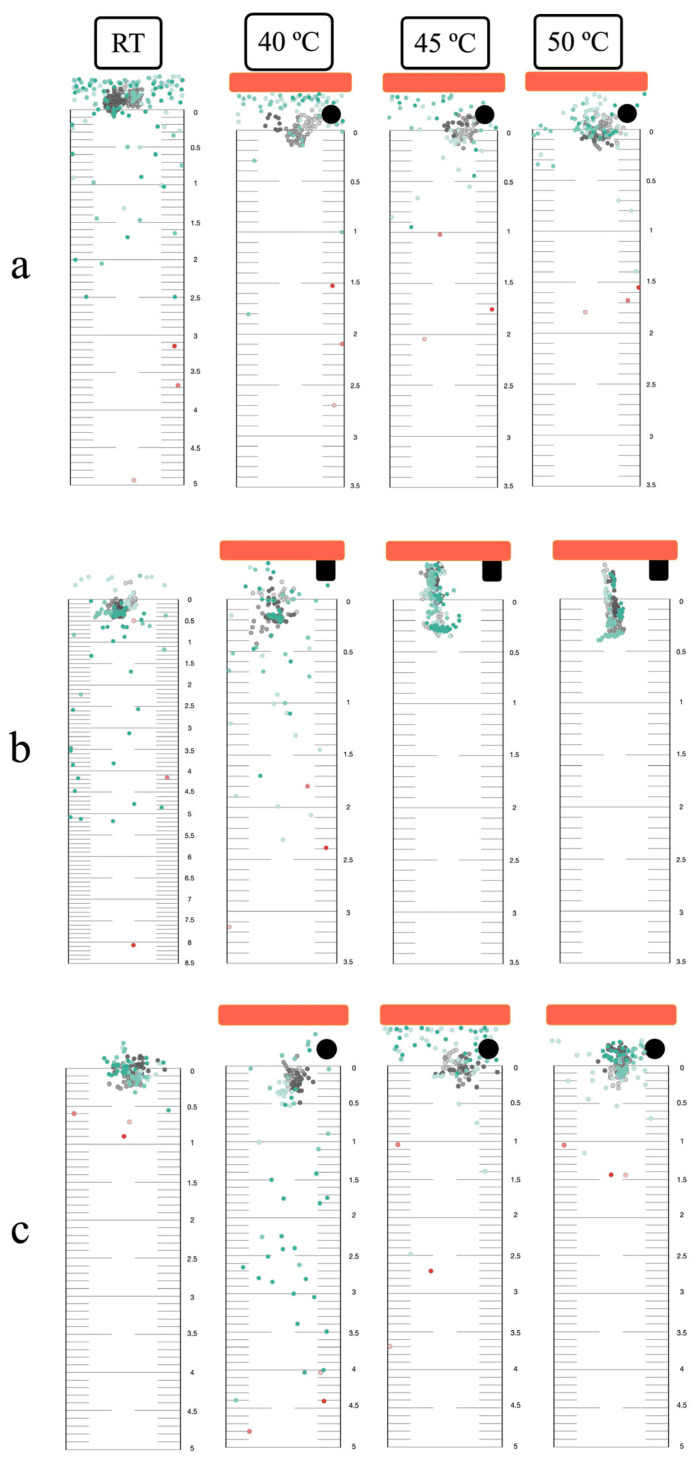
Group vertical migration response to noxious heat: (**a**) Surface route with hard thermotaxis agar and open chamber. (**b**) Within-agar route with semi-solid thermotaxis agar and closed chamber. (**c**) Mixed migration route with semi-solid thermotaxis agar and open chamber. Orange bars: the radiant heater. Black circles or rectangles: NTC temperature sensors. Gray dots: starting position. Green dots: position at the end of observation. Red dots: the longest downward migratory distance of each replicate. All experiments were conducted in three replicates.

At room temperature, single larvae exhibited downward localized searching in all migration assays (Figure 6). Group migration, however, showed reduced localized searching in the mixed migration assay (Figure 7 and Figure S3). At 40 °C, single larvae displayed both upward positive thermotaxis and downward localized searching across all migration routes. Prominent positive thermotaxis was exhibited in group migration at 40 °C for the surface migration assay, while within-agar and mixed migration showed increased downward localized searching. At 45 °C, single larvae exhibited increased localized searching in surface and mixed migration assays. Within-agar migration at 45 °C showed continued larval survival, increased downward localized searching, and a lack of complete inactivation, contrasting with observations in the horizontal plane (Figure 6b). Complete inactivation was observed in group within-agar migration assays at both 45 °C and 50 °C (Figure 7b). Surface and mixed group migration assays at these temperatures showed increased positive thermotaxis and incomplete inactivation (Figure 7a,c).

### 3.6. Long-Range Attraction and Response to Noxious Heat

**Figure 8:**
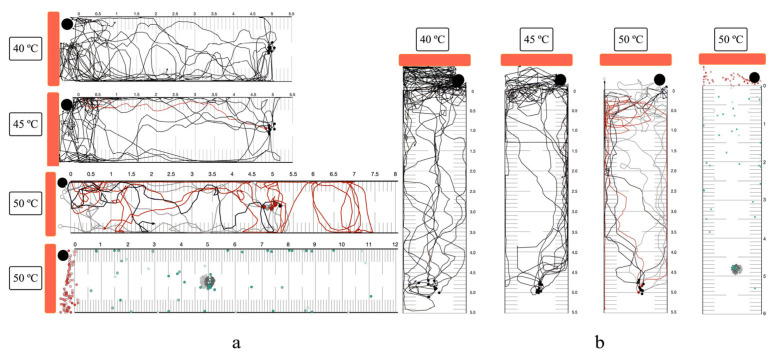
Long-range noxious heat attraction of *S. ratti* infective larvae. (**a**) Horizontal orientation. (**b**) Vertical orientation. The red line represents the migratory track of a single worm that evades the heat source. Orange bars: the radiant heater. Black circles or rectangles: NTC temperature sensors. Black dots and arrows: larval positive thermotaxis. Red dots and arrows: larval negative thermotaxis. The gray line with an empty circle endpoint and a red dot represent the migratory track and position of inactivated larvae for single-worm migration and group migration. Gray dots: starting position of migration. Green dots: position at the end of observation. The single-worm migration assay was conducted in ten replicates, and the group migration assay was conducted in three replicates.

In both horizontal and vertical planes, nearly all single larvae exhibited positive thermotaxis, migrating beyond the 0 cm line at 40 °C and 45 °C (Figure 8). One larva in the horizontal plane at 45 °C turned before reaching the 0 cm mark. Upon entering the lethal heat zone at 50 °C, single larvae showed a lack of negative thermotaxis and were inactivated. Inactivation rates at 50 °C were 53.38% in the horizontal plane and 43.87% in the vertical plane. The starting point temperatures for 40 °C, 45 °C, and 50 °C, measured for horizontal and vertical planes, were 26.2 °C/26.1, 26.4 °C/26.2 °C, and 26.5 °C/26.4 °C, respectively.

### 3.7. Heat Attraction and High Concentration Saline Trapping

**Figure 9:**
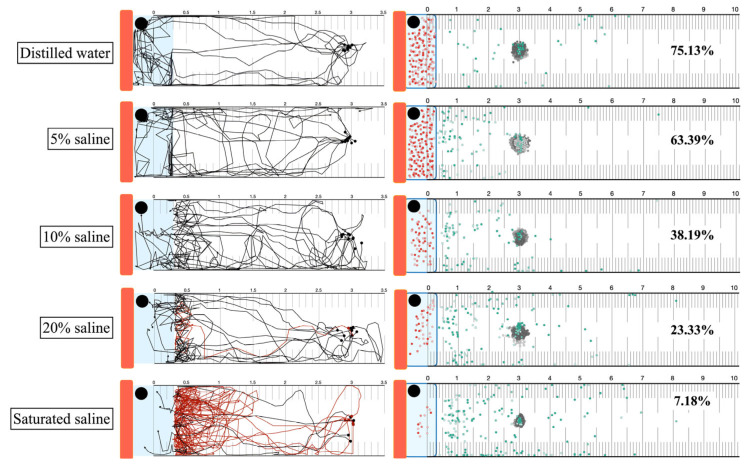
Attraction of *Strongyloides* larvae to high-saline traps under heat stimulus. Orange bars: the radiant heater. Black circles: NTC temperature sensors. Blue rectangles represent saline areas, and blue rectangles with borders represent saline wells. The red line represents the migratory track of a single worm that was repelled from the saline. Black dots and arrows: larval positive thermotaxis. Red dot: number of larvae trapped in saline well (not exact position). Gray dots: starting position of migration. Green dots: position at the end of observation. The single-worm migration assay was conducted in ten replicates, and the group migration assay was conducted in three replicates.

Larvae exhibited positive thermotaxis towards the heat source, subsequently contacting various saline concentrations. Larval behavior upon contact was categorized as entry, avoidance, or hesitation (Figure 9 and Figure S4). In distilled water and 5% saline, larvae migrated directly into the liquid area. The highest frequency of hesitation and avoidance was observed upon contact with saturated saline. The percentage of larvae trapped within the liquid wells was calculated from group migration assays with 75.13% for distilled water, 63.39% for 5% saline, 38.19% for 10% saline, 23.33% for 20% saline, and 7.18% for saturated saline. The starting point temperature was 28.1 °C.

### 3.8. Thermal and Saline Agar Trapping for S. ratti Larvae from Soil

**Figure 10:**
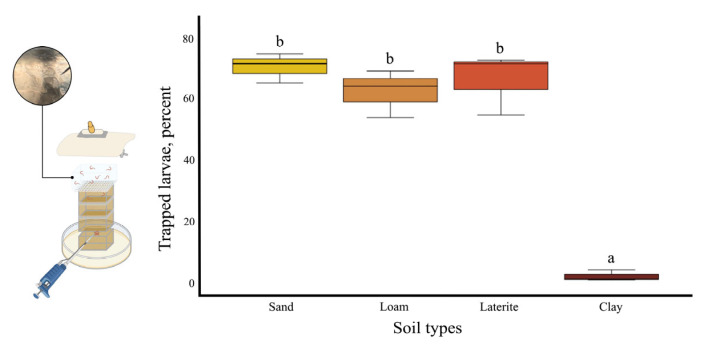
Heat–saline agar trapping for four soil types. All experiments were conducted in three replicates. Different letters (a, b) indicate statistically significant differences between groups based on the Tukey HSD post hoc test.

The number of infective larvae migrating from a 3 cm soil depth to the topsoil and into the agar cuboid was quantified to assess larval attraction by heat, moisture, and gravity. The lure ratio, calculated as the number of larvae recovered from agar and topsoil divided by the total the total number of larvae initially placed, was 0.82 for sand, 0.80 for loam, 0.76 for laterite, and 0.03 for clay. The agar-to-topsoil ratio, representing the efficiency of the agar cuboid in trapping surface larvae, was calculated by dividing the number of larvae trapped in the agar by the number of larvae recovered from agar and topsoil. This ratio was similar across all soil types. The overall trapping effectiveness, representing the percentage of initially placed larvae recovered in the salted agar cuboid, was 70.87 ± 2.80% for sand, 62.73 ± 4.52% for loam, 66.69 ± 5.84% for laterite, and 2 ± 1.09% for clay (Figure 10).

## 4. Discussion

In this study, *S. stercoralis* infective larvae, obtained from clinical specimens, were used for long-term mortality assessments, while *S. ratti* larvae were employed for detailed short-term exposure experiments. For thermal inactivation for *Strongyloides* larvae, we observed that long-term incubation at 50 °C for 12 or 24 h, as well as short-term incubation at 50 °C for 20 min, resulted in significant larval mortality. These findings diverge from the study of larvae inactivation in fecal pit soil that required 60 °C with 60 min of exposure [33]. This discrepancy may be attributed to differences in experimental setup, particularly the media type and volume (10 g of fecal pit soil versus 50 μL of distilled water in our study). Soil with a high organic content likely exhibits greater thermal inertia (thermal conductivity of water = 0.57 W/m·K, organic matter = 0.25 W/m·K), requiring higher temperatures and longer exposure times for effective heat conduction and subsequent larval inactivation [44]. We observed minimal larval inactivation across a pH range of 3 to 11, with mortality rates of approximately 5% for long-term exposure of *S. stercoralis* and inactivation of approximately 7% for short-term exposure of *S. ratti*. This contrasts with findings from a reclaimed wastewater treatment study, where significant larval mortality was reported at pH 10.2 using sodium hydroxide [34]. Similar results were found in the nematocidal effect of pH on *S. papillosus*, which required low pH (pH 2.2 using formic acid) and high pH (pH 13 using sodium hydroxide) with 24 h incubation [45,46]. This discrepancy warrants further investigation, particularly considering the known adaptability of *Strongyloides* larvae to extreme environmental changes. Potential mechanisms contributing to this resilience include the protective function of the larval cuticle against corrosive chemicals, the presence of an internal buffering system to maintain intracellular pH homeostasis, and the adaptation of enzymes to neutralize oxidative stress induced by strong alkaline conditions [47,48]. In this study, 9% saline concentration resulted in the inactivation of *S. ratti* larvae after 50 min of incubation. This concentration requirement is lower than that reported for *S. stercoralis* immobilization (3 M NaCl, equivalent to 17.55% saline) in a chemotaxis assay involving 5 min of contact [45], and also lower than the saturated salt solution required for killing hookworm filariform larvae after 30 min of contact [49]. The application of combined stress, using 40 °C and 4% saline, effectively reduced the individual requirements for heat (50 °C) and saline (9% saline) to achieve larval inactivation within 50 min. This observation suggests a synergistic effect, wherein the combined factors accelerate protein denaturation, induce water loss, and disrupt cellular mechanisms [50,51].

The particle size distribution of the four dried soil samples, from largest to smallest, was as follows: sand, loam ≈ laterite, and clay. Sand, characterized by its high quartz content, exhibited the highest heat conduction. In contrast, dried clay, with its lower quartz content and light, loose density, demonstrated lower heat conduction and the slowest rate of larval inactivation [52].

The thermotactic response of infective larvae to noxious heat was examined, exceeding the physiological limits reported for free-living nematodes (15–25 °C) and the upper temperature limit previously used for *Strongyloides* studies (>40 °C) [36,53]. Utilizing a transparent agar environment facilitated detailed tracking of larval movement, providing a proxy for behavior in soil. At 40 °C, larvae exhibited positive thermotaxis towards the heat source. This behavior aligns with the known role of amphid sensory neurons (specifically *Strongyloides* ALD) in heat sensing for host seeking [36]. The biphasic calcium response activity of ALD, distinct from the *Caenorhabditis elegans* AFD homolog, involves an initial inhibition of excitation upon contact with temperature above ambient, followed by release of inhibition near host skin temperature [54]. At 45 °C, larvae displayed a slight movement away from the heat source, accompanied by increased localized searching. Unlike free-living nematodes, the negative thermotaxis in *Strongyloides* is not solely attributed to the recognition of heat as a danger signal. Instead, it likely reflects a strategy to migrate towards cooler regions, thereby increasing the probability of encountering a host (improve detection of the thermal gradient between host and environment) [55]. Upon exposure to lethal heat (50 °C), immediate inactivation was observed in all single-worm assays. This was likely due to the time required for water vaporization (2–3 min) before larvae could migrate out of the heated area, resulting in complete thermal inactivation.

Larval migration routes within the high-temperature agar environment were affected by both the internal energy (an extensive property) and temperature (an intensive property) of the media [56,57]. When exposed to a uniform high temperature (45 °C or 50 °C) at both agar surface and within the agar, the preferred migration route of larvae in this condition was on the agar surface. This preference likely stems from sensing less heat compared to movement within the agar matrix. Consequently, larvae migrated upwards to the agar surface and remained viable in the open-chamber semi-solid agar thermotaxis assay, while complete inactivation was observed in the closed-chamber assay. However, a notable exception was observed in the single-worm vertical migration assay conducted in a closed chamber with semi-solid agar at 45 °C, where larvae did not exhibit complete inactivation, in contrast to group migration assays under the same experimental conditions. This unexpected phenomenon warrants further investigation to elucidate the underlying mechanisms.

The observed contrast between single-worm and group migration results may be attributed to inter-larval communication in response to stimuli. Studies on entomopathogenic nematodes have documented group communication in the absence of external stimuli, as well as track-following behavior [58,59]. While this phenomenon is not well-characterized in mammalian parasitic nematodes, our incidental observation during single-worm migration assays revealed that a second *Strongyloides* larva, placed sequentially, tended to follow the track of the preceding larva towards the heat stimulus.

The long-range migration assay confirmed that larvae exhibit a strong positive thermotactic response towards intense heat stimuli, providing an opportunity for targeted inactivation within the lethal heat zone. Notably, this assay also revealed that a substantial proportion (up to half) of the larval population was inactivated by a false positive thermal source (non-host heat). Furthermore, viable larvae that were not immediately inactivated often reached a turning point very close to the rim of the lethal heat zone, suggesting a potential for behavioral avoidance or a threshold response to the intense heat. The precise temperature at which larval migration reverses in response to a supra-physiological heat source should be evaluated to determine and add up with the previously reported turning point at the end of non-host heat gradient below 30 °C [53].

The strong chemical repellence of 10% saline to saturated saline solutions (distinct hesitation track from single worm migration assay) overcame 40 °C heat attraction, indicating that the hypothesized thermal-high concentrate saline traps were not effective. The thermal and saline agar trapping assay was designed to demonstrate the removal of mammal parasitic nematode larvae from soil, using minimal heat and salt concentrations to target these parasites while minimizing potential harm to non-target organisms such as free-living nematodes, entomopathogenic nematodes, and plants. While cattle parasitic nematodes can migrate vertically through soil for distances of 25 cm upward and 30 cm downward, the uppermost soil layer, which is in direct contact with skin, poses the highest risk of infection [60,61,62]. In our study, we observed that larvae sensed a heat source from 3 cm and subsequently increased their movement speed towards it in both horizontal and vertical planes. Rehydrating the soil samples to 10% water content enhanced heat conduction by facilitating water bridging between soil particles [42]. Added warm distilled water to the topsoil surface also prevented premature drying of the soil surface during heating, which could impede larval penetration. Moisture and saline gradients were established by adding water and through release from the cuboid agar. Larvae migrating upwards were inactivated within the agar matrix, likely due to increased internal energy and the presence of 5% saline. In contrast, larvae migrating on the agar surface exhibited slow movement. The lure ratio indicated that clay soil was the least effective for heat attraction, despite exhibiting a similar agar-to-topsoil larvae ratio compared to other soil types. This finding aligns with previous research demonstrating restricted vertical movement of cattle parasitic nematodes through heavy clay soils [63]. Sand exhibited the highest effectiveness for larval trapping, likely due to its superior heat conduction. Once trapped, larvae could be readily eliminated through heat or chemical treatment.

## 5. Conclusions

This study demonstrated the effectiveness of 50 °C for inactivating *Strongyloides* infective larvae through both long-term and short-term exposures. A combined treatment of 40 °C and 4% saline achieved larval inactivation within 50 min. Exposure to noxious heat elicited distinct behavioral responses: positive thermotaxis at 40 °C, localized searching at 45 °C, and complete inactivation at 50 °C. Larval inactivation was observed within semi-solid agar during within-agar migration at 45 °C. Long-range heat attraction using 50 °C resulted in 40% inactivation in the vertical plane and 50% inactivation in the horizontal plane. The combined heat attraction and high-concentration saline trapping method proved ineffective. Sand was identified as the most effective soil type for heat conduction and heat–saline agar trapping, while clay was the least effective. These findings suggest the potential for developing heat-attraction trapping methods for larger-scale devices.

## Figures and Tables

**Figure A1:**
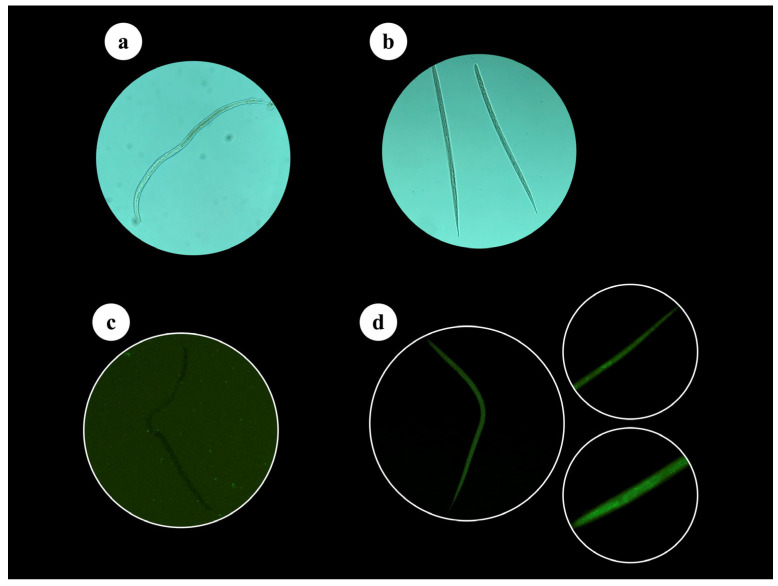
Long-term temperature and pH-treated *S. stercoralis* infective larvae. Bright field 100×: (**a**) Active and viable larvae with preserved esophageal-intestine morphology. (**b**) Dead larvae with straight posture and degraded esophageal-intestine morphology. Florescent mode 100×: (**c**) Viable larva, unstained. (**d**) Dead larva with nuclei stained throughout the worm’s body.

**Figure A2:**
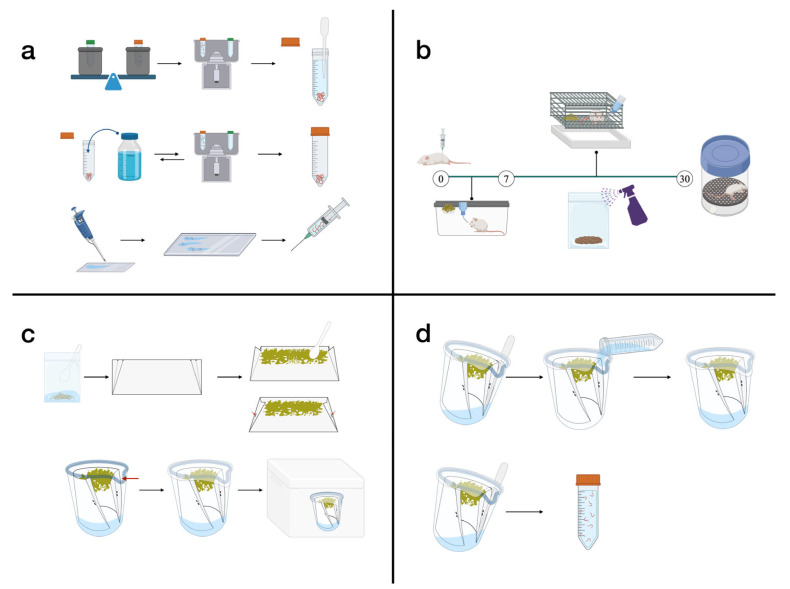
Maintenance of *S. ratti* life cycle. (**a**) Washing step with antibiotic to obtain clean larvae for injection. (**b**) Animal rearing in the animal unit. (**c**) Stool culture using modified Harada–Mori filter paper technique. (**d**) Infective larvae collection.

## Data Availability

The datasets used and/or analyzed during the current study are available from the corresponding author upon reasonable request.

## Supplementary Materials

The following supporting information can be downloaded at: https://www.mdpi.com/article/10.3390/biology14050559/s1.

## Appendix A

### Appendix A.1. Propidium Iodide Staining and Larvae Appearance

Propidium iodide (PI) solution (Invitrogen^®^ by Thermo Fisher Scientific, Hillsboro, OR, USA) was prepared by dissolving 100 mg of PI in 100 mL of deionized water to obtain a final concentration of 1 mg/mL. For staining, 50 μL of PI solution was added to each larval sample well and incubated for 20 min in the dark [64,65].

The movement and mortality of the worms were assessed using a confocal laser scanning microscope (ZEISS^®^ LSM800 by Carl Zeiss Microscopy GmBH, Oberkochen, Germancy). Larval movement and gross morphology were observed under 100× magnification in bright-field mode. Mortality was determined by switching to fluorescence mode to visualize fluorescent labeling throughout the worm body, including the hypodermal nuclei, esophageal nuclei, intestinal nuclei, and tail nuclei (Figure A1).

### Appendix A.2. Maintenance of Strongyloides ratti Life Cycle

*Strongyloides ratti* infective larvae (iL3) were collected from the culture water of the primary project of Prof. Paiboon Sithithaworn. The larvae were washed using penicillin–streptomycin antibiotic solution (100 units of penicillin/mL) through centrifugation at 3000 rpm, 4 °C, for 10 min, repeated for three cycles (Figure A2a). Male Wistar rats (4–6 weeks old) were sedated in a 5 L sedation jar containing a cotton pad soaked with 1 mL of isoflurane placed under a metallic stage (2% isoflurane). Once the rats reached optimal sedation, 70% alcohol was sprayed onto the posterior back, and 4500 washed larvae were injected subcutaneously. Infected rats were housed in the animal facility at the Faculty of Medicine, Khon Kaen University. Seven days post-infection, the rats were transferred to hanging cages for rearing, and fecal samples were collected every day until 30 days post-infection. The rats were euthanized by 4% isoflurane (Figure A2b).

Stool cultures were performed daily using a modified Harada–Mori filter paper culture technique. Grade 1 qualitative filter paper (Whatman^®^) was selected and appropriately cut for suitability in water collection and rehydration during the culture process. The filter papers were cut into strips measuring 23 × 10 cm, with folds of 3 cm and 1.5 cm prepared at both ends for ease of use. Rat fecal samples were soaked in water for at least 3 h before being crushed, mixed, and spread evenly on the filter paper. The prepared culture papers were then stapled and placed into a 500 mL beaker containing 50 mL of filtered water, ensuring paper’s folded ends were positioned at the pouring site to facilitate water movement. The setup was covered with plastic wrap to maintain humidity and labeled with the culture date, mixed-stage larvae collection date, and infective larvae collection date, and then placed in a foam box at room temperature (25 °C) (Figure A2c). Mixed-stage larvae were discarded, and infective larvae were collected on the fifth day of culture (Figure A2d).

### Appendix A.3. Prepare Thermotaxis Agar

Hard agar, 4.25 g of pure agar, and 0.75 g of NaCl were dissolved in 250 mL of distilled water, autoclaved at 121 °C for 20 min, and allowed to cool to 55 °C. Then, 0.25 mL of 1 M CaCl_2_, 0.25 mL of 1 M MgSO_4_, and 6.25 mL of 1 M KH_2_PO_4_ (pH 6.0) were added [66]. Semi-solid agar, 1 g of pure agar, and 0.75 g of NaCl were dissolved in 250 mL of distilled water, autoclaved at 121 °C for 20 min, and allowed to cool to 55 °C. Then, 0.25 mL of 1 M CaCl_2_, 0.25 mL of 1 M MgSO_4_, and 6.25 mL of 1 M KH_2_PO_4_ (pH 6.0) were added [67,68]. Sealant agar, 5 g of pure agar, was dissolved in 100 mL of distilled water and autoclaved at 121 °C for 20 min.
