# Supplementary material for: Tricky with Heat and Salt: Soil Factors, Thermotaxis, and Potential for Heat–Saline Agar Trapping of Strongyloides Larvae
Source: Biology (Basel). 2025 May 16;14(5):559. doi: 10.3390/biology14050559 (PMC12109420; doi:10.3390/biology14050559)
Supplement: Supplementary file 1 [file biology-14-00559-s001.zip › biology-3602752-Supplementary Materials.pdf]

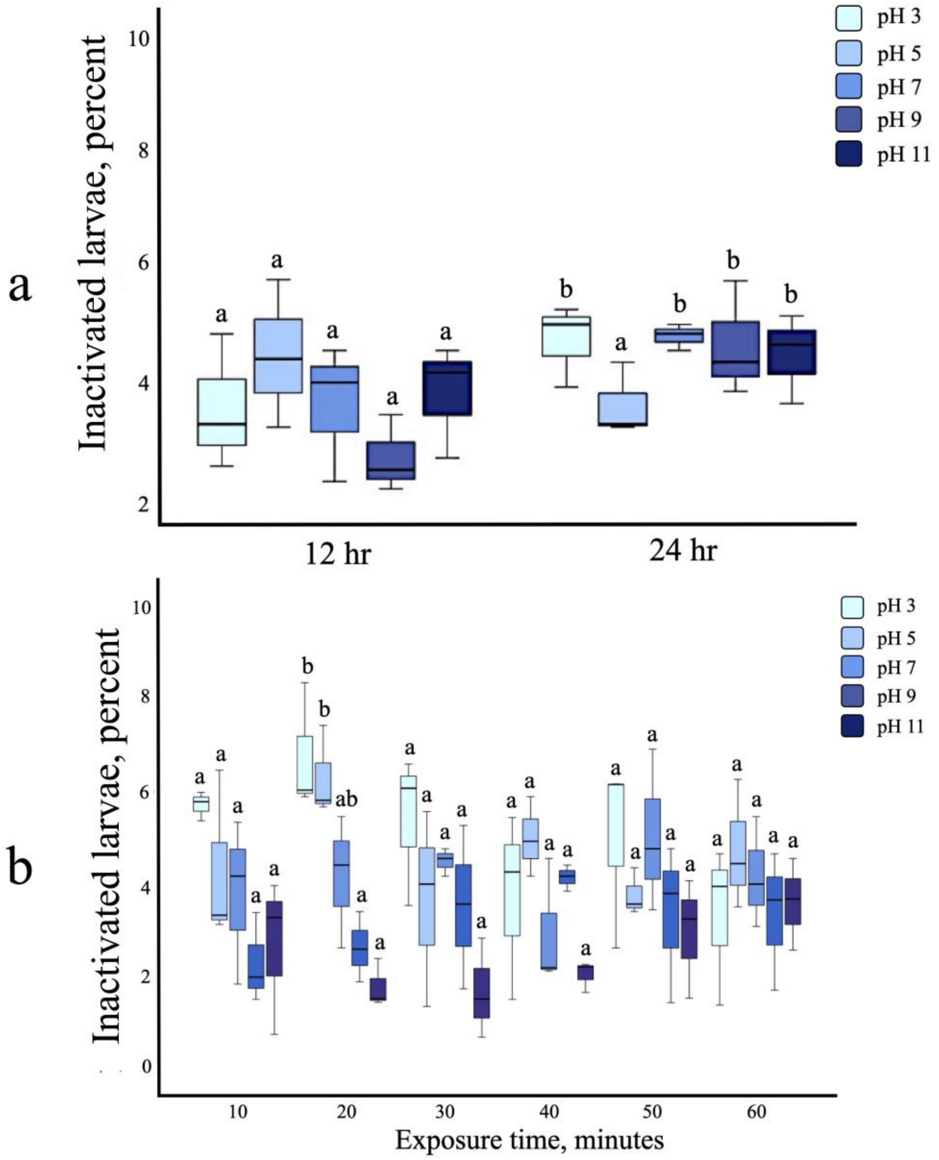

Figure S1. Effect of pH exposure on *S. stercoralis* and *S. ratti*.: (a) Long–term exposure of *S. stercoralis* iL3 to pH3 to pH11; (b) Short–term exposure of *S. ratti* iL3 at pH3 to pH11. All experiments were conducted in three replicates. Different letters (a, b, c, d) indicate statistically significant differences between groups based on the Tukey HSD post-hoc test.

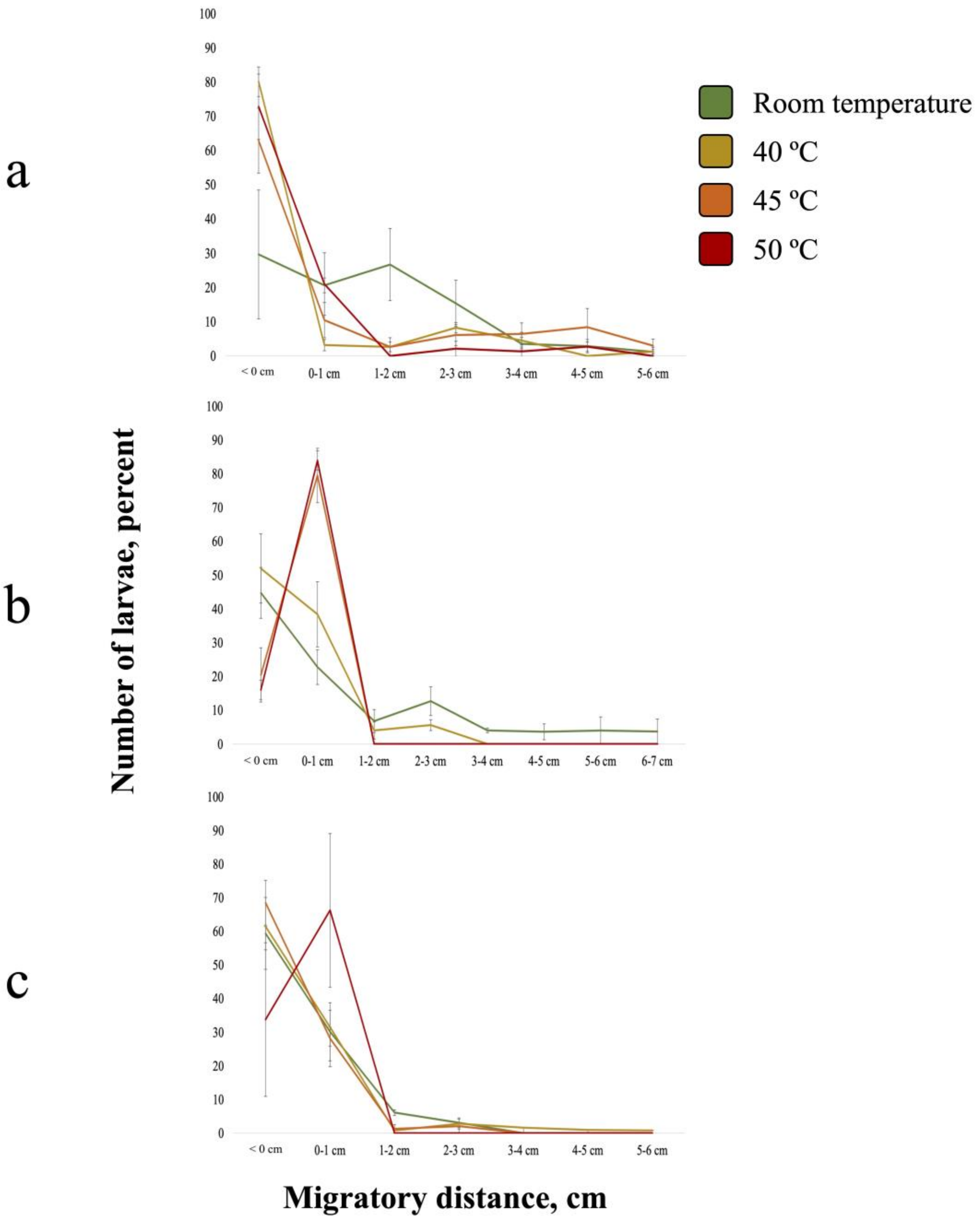

Figure S2. Larval horizontal distribution across temperatures: (a) Surface route with hard thermotaxis agar and open chamber; (b) Within agar route with semi–solid thermotaxis agar and closed chamber; (c) Mixed migration route with semi–solid thermotaxis agar and open chamber. Green line – room temperature; yellow line – 40°C; orange line – 45°C; red line – 50°C. All experiments were conducted in three replicates. Data on infective larval distribution were analyzed and presented as mean ± standard error of mean (SEM).

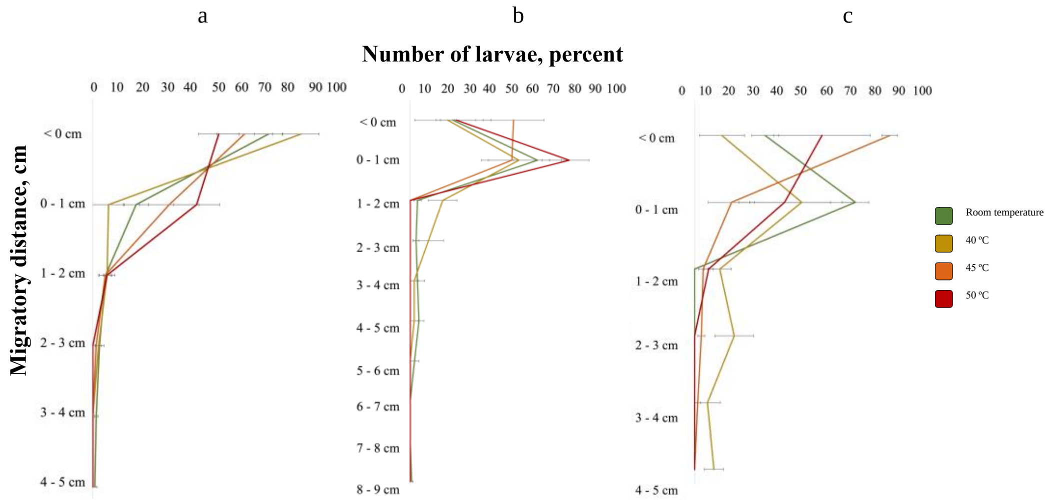

Figure S3. Larval vertical distribution across temperatures: (a) Surface route with hard thermotaxis agar and open chamber; (b) Within agar route with semi–solid thermotaxis agar and closed chamber; (c) Mixed migration route with semi–solid thermotaxis agar and open chamber. Green line – room temperature; yellow line – 40°C; orange line – 45°C; red line – 50°C. All experiments were conducted in three replicates. Data on infective larval distribution were analyzed and presented as mean ± standard error of mean (SEM).

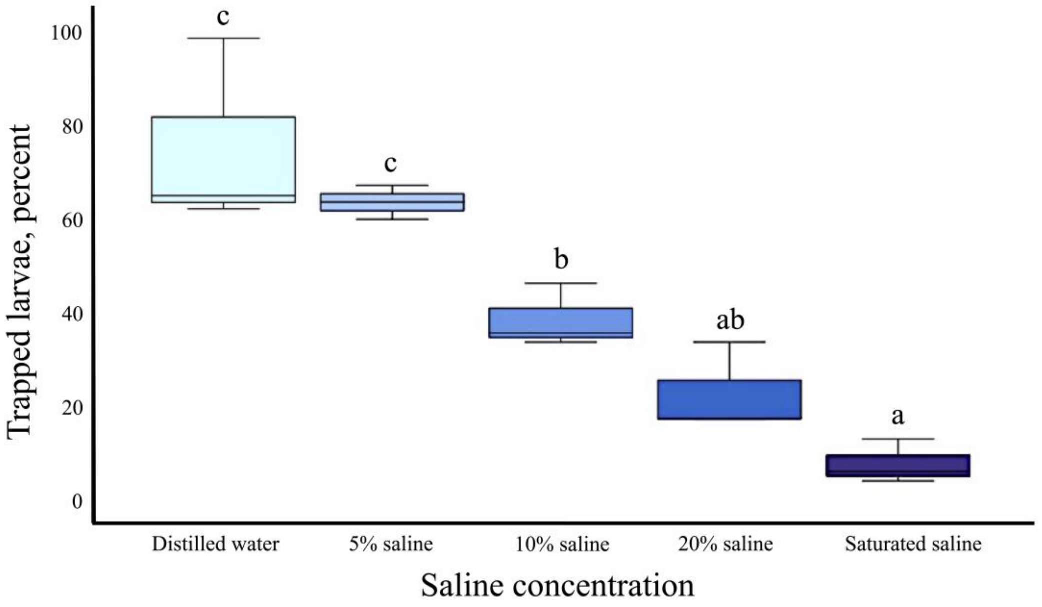

Figure S4. Attraction of *Strongyloides* larvae to high saline-well traps under heat stimulus. All experiments were conducted in three replicates. Different letters (a, b, c, d) indicate statistically significant differences between groups based on the Tukey HSD post-hoc test.
